# Continuous enzyme activity assay for high-throughput classification of histone deacetylase 8 inhibitors

**DOI:** 10.37349/etat.2023.00144

**Published:** 2023-06-30

**Authors:** Markus Schweipert, Anuja Amurthavasan, Franz-Josef Meyer-Almes

**Affiliations:** University of Southampton, UK; Chemical Engineering and Biotechnology, University of Applied Sciences Darmstadt, 64295 Darmstadt, Germany

**Keywords:** Continuous enzyme activity assay, histone deacetylases, binding mode, binding mechanism, adverse effects, high-throughput screening

## Abstract

**Aim:**

Human histone deacetylase 8 (KDAC8) is a well-recognized pharmaceutical target in Cornelia de Lange syndrome and different types of cancer, particularly childhood neuroblastoma. Several classes of chemotypes have been identified, which interfere with the enzyme activity of KDAC8. These compounds have been identified under equilibrium or near equilibrium conditions for inhibitor binding to the target enzyme. This study aims for the classification of KDAC8 inhibitors according to the mode of action and identification of most promising lead compounds for drug development.

**Methods:**

A continuous enzyme activity assay is used to monitor inhibition kinetics.

**Results:**

A high-throughput continuous KDAC8 activity assay is developed that provides additional mechanistic information about enzyme inhibition enabling the classification of KDAC8 inhibitors according to their mode of action. Fast reversible inhibitors act as a molecular chaperone and are capable to rescue the enzyme activity of misfolded KDAC8, while covalent inactivators and slow dissociating inhibitors do not preserve KDAC8 activity.

**Conclusions:**

The application of continuous KDAC8 activity assay reveals additional information about the mode of interaction with inhibitors, which can be used to classify KDAC8 inhibitors according to their mode of action. The approach is compatible with the high-throughput screening of compound libraries. Fast reversible inhibitors of KDAC8 act as molecular chaperones and recover enzyme activity from misfolded protein conformations. In contrast, slow-binding inhibitors and covalent inactivators of KDAC8 are not capable to recover enzyme activity.

## Introduction

**Figure 1:**
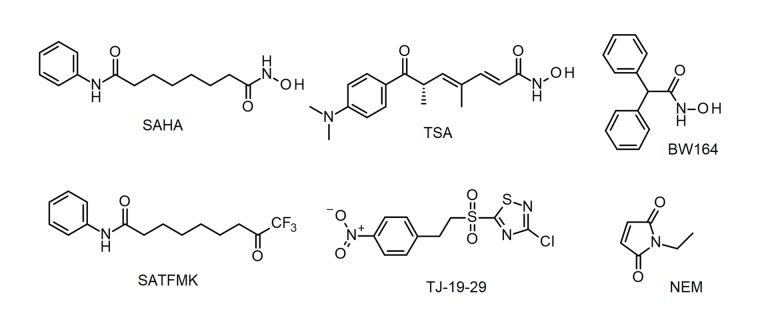
KDAC8 inhibitors used in this study. BW164: 2,2-diphenylethanehydroxamic acid; NEM: *N*-ethylmaleimide; SAHA: suberoylanilide hydroxamic acid; SATFMK: 9,9-trifluoro-8-oxo-*N*-phenyl-nonanamide; TJ-19-29: 3-chloro-5-[2-(4-nitrophenyl)ethanesulfonyl]-1,2,4-thiadiazole

Human histone deacetylases constitute a family of enzymes, which catalyze the removal of acetate or acyl modifications from the ε-amino group of lysine residues in histones or other target proteins. Since the number of discovered non-histone substrates is continuously growing, these enzymes are more correctly designated as lysine deacetylases/deacylases (KDACs). KDACs are involved in many important physiological and pathophysiological processes. Consequently, KDACs play important roles in multiple diseases, especially different types of cancer [1]. KDACs are divided into 4 classes based on sequence and functional similarities. Class III contains nicotinamide adenine dinucleotide (NAD^+^)-dependent KDACs, whereas all other classes consist of Zn^2+^-dependent enzymes. Despite belonging to class I, human histone deacetylase 8 (KDAC8) stands apart from other members by not participating in multi-protein complexes that are typical for class I and II KDACs, and also exhibiting unique substrate specificities. For example, the preferential cellular substrates of KDAC8 are not histones, but acetylated structural maintenance of chromosomes 3 (SMC3), estrogen-related receptor-α (ERR-α), retinoic acid-induced 1 (RAI1), myeloid/lymphoid or mixed-lineage leukemia 2 (MLL2), p53, and cortactin [2–5]. Human KDAC8 is an established target in childhood neuroblastoma and is also discussed in other tumor indication areas such as breast cancer and urothelial cancers [6, 7]. Several structurally diverse KDAC8 inhibitors have been described, which target the malleable binding pocket, acetate release channel, and surface of the enzyme (representative examples in Figure 1) [8–14]. The adaptability of L1, L2, and L6 loops allows for the accommodation of various pharmacophore structures, and facilitates the creation of a temporary side pocket, as demonstrated by the crystal structure of KDAC8 bound with trichostatin A (TSA), where an additional inhibitor molecule is located in this pocket near the primary catalytic binding site [Protein Data Bank (PDB)-ID: 1T64]. The different conformations of KDAC8 in complex with different ligands suggest different and complex binding mechanisms and may have consequences for kinetic features like residence time and transforming the binding event into the biological response or the intended pharmacological effect.

Nowadays, drug discovery is a highly industrialized process, which relies on high-throughput screening and other techniques resulting in a very high number of putative lead compounds. However, most of these compounds, fail in pre-clinical or the first clinical phase due to unexpected adverse effects causing a high attrition rate and immense development costs. Therefore, it is highly desired to employ efficient methods to stratify and identify the most effective substances at the earliest possible stage to concentrate the resources on the most promising candidates for drug development. For this purpose, several metrics, based on physicochemical properties, are used to assess the drug likeliness of lead compounds, and a variety of assays are performed to assess the accompanied adsorption, distribution, metabolism, excretion (ADME) properties and potential toxicity. Furthermore, it is known that the kinetics and mechanisms by which effective compounds exert their effect are important for the transmission of the initial target binding signal into a biological or pharmacological response. Therefore, high-throughput kinetic methods and the deduction of mechanistic information are paramount to understanding the mode of action of an enzyme inhibitor. Here, a high-throughput and quantitative kinetic method is described to determine mechanistic features of KDAC8 inhibition allowing for quick classification of different types of inhibitors. This is an invaluable addition to existing metrics to come too early decisions on the most promising lead structures, which should be taken further in the drug development process.

## Materials and methods

### Recombinant production of KDAC8

The method employed has been described previously [15]. In short, full-length human KDAC8 was produced in *E. coli* BL21 (DE3) (New England BioLabs^®^, Ipswich, MA, USA) with a pET14b vector system (Novagen^®^, EMD Millipore, Burlington, MA, USA) containing the *KDAC8* gene fused to a N-terminal His6-small ubiquitin-like modifier (SUMO) tag using autoinduction growth media (3.08 g/L KH_2_PO_4_, 3.1 g/L Na_2_HPO_4_ * 2 H_2_O, 0.44 g/L MgSO_4_ * 7 H_2_O, 0.1% lactose, 0.05% glucose, 0.5% glycerol, and 20 g/L Lennox LB media). Purification was performed by immobilized-metal affinity chromatography (IMAC), and the His6-SUMO tag was removed using SUMO protease. Further purification steps were ion exchange chromatography and size exclusion chromatography.

### Determination of inhibitor concentration that inhibits 50% of enzyme activity values

For the continuous method, a serial dilution of the respective ligand in assay buffer (25 mmol/L Tris-HCl, 75 mmol/L KCl, 0.00001% Pluronic, pH 8.0) was incubated with 10 nmol/L KDAC8 in a black 96 well half area microtiter plate (Greiner Bio-One, Solingen, Germany) for 1 h at 30°C. The enzymatic reaction was initiated by the addition of Master Mix: 20 µmol/L tert-butyl *N*-[(1S)-1-[(4-methyl-2-oxo-chromen-7-yl)carbamoyl]-5-[(2,2,2-trifluoroacetyl)amino]pentyl]carbamate, commonly known as Boc-Lys(TFA)-AMC substrate (Bachem, Bubendorf, Switzerland), and 0.1 mg/mL trypsin (AppliChem). The temporal release of 7-amino-4-methyl-coumarin (AMC) was observed in a microplate reader (Spark^®^, Tecan) at 460 nm (Ex: 340 nm). The data points were plotted against time and blank corrected. For inhibitor concentration that inhibits 50% of enzyme activity (IC_50_) determination a linear regression analysis in the initial slope (*v_i_*) of each curve was performed and normalized using a positive control without ligand. The obtained normalized enzyme activities were plotted against the respective ligand concentration and fitted with a four-parameter logistic function using GraphPad Prism software:

**Figure:**
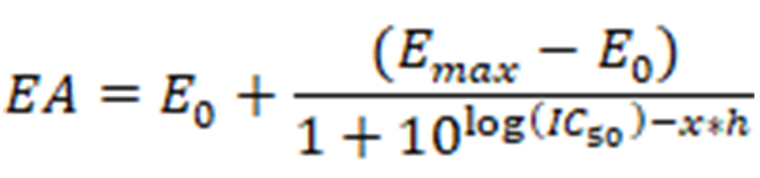


where *EA* stands for the enzyme activity at a given ligand concentration *x*, *E_max_* and *E_0_* are the enzyme activities without ligand and under complete inhibition, respectively. *IC_50_* is the ligand concentration at which half of the enzyme activity is inhibited and *h* is a measure of the steepness of the dose-response curve. For the determination of time-dependent IC_50_ values with KDAC8, the basic procedure was identical, but different pre-incubation times for protein and ligands were used (5 min to 60 min) as well as 100 nmol/L KDAC8 instead of 10 nmol/L KDAC8. All solutions were pre-incubated at 30°C. The two-step IC_50_ determination has been described previously [16]. In short: KDAC8 was incubated with the respective ligands, and the enzymatic reaction was started by the addition of 20 µmol/L Boc-Lys(TFA)-AMC substrate (Bachem). After another incubation step the enzymatic reaction was terminated by the addition of 1.7 µmol/L SATFMK. Fluorescent AMC was released by the addition of 0.4 mg/mL trypsin. In this endpoint assay, the fluorescence signal was correlated to KDAC8 activity and normalized with a positive control without ligand. Data analysis was performed as described above. For IC_50_ determination due to trypsin inhibition, the basic IC_50_ procedure was identical. In the assay 0.1 mg/mL trypsin and 400 µmol/L Bz-Arg-PNA (Bachem) were used. The change in substrate absorbance was recorded in a microplate reader (Spark^®^, Tecan) at 405 nm. In this endpoint assay, the absorbance signal was correlated to KDAC8 activity and normalized with a positive control without ligand. Data analysis was performed as described above.

### Determination of Michaelis-Menten parameters

The measurements were carried out in a black 96-well microtiter plate (Greiner) at 30°C in assay buffer (see above). A serial two-fold dilution ranging from 50 µmol/L substrate, Boc-Lys(TFA)-AMC (Bachem), to 780 nmol/L, was prepared in an assay buffer containing 0.1 mg/mL trypsin. The enzymatic reaction was initiated by the addition of 10 nmol/L KDAC8. The release of fluorogenic AMC was observed in a micro plate reader (PHERAstar Optima, BMG Labtech) at 450 nm (Ex: 350 nm). The data points were plotted against time and blank corrected. For the determination of Michaelis-Menten parameters, a linear regression analysis of the initial slope (*v_i_*) of each curve was performed. Slopes were transformed into rates of AMC product formation using a calibration curve with different AMC concentrations. The obtained enzyme conversion rates were plotted against the respective substrate concentration and fitted to the Michaelis-Menten function using GraphPad Prism software. The turnover number (*k_cat_*) was calculated as

**Figure:**
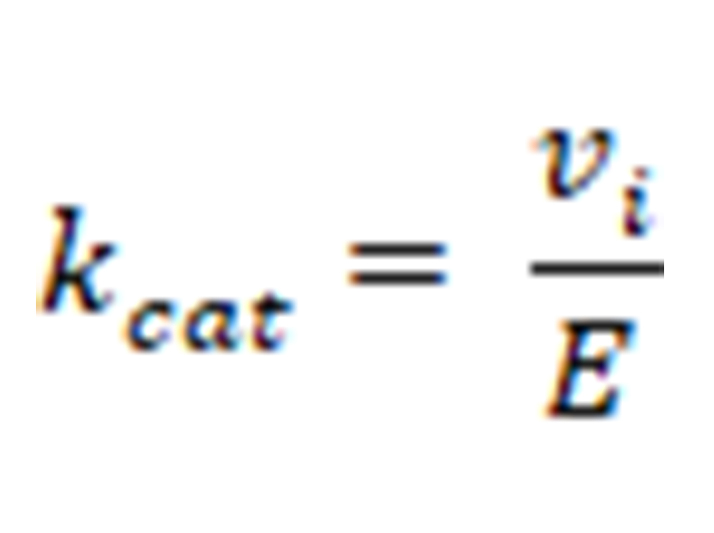


where *v_i_* is the substrate conversion rate and *E* is the enzyme concentration. All solutions were pre-incubated at 30°C.

### Analysis of progress curves of enzyme reaction

The measurements were carried out in a black 96-well half-area microtiter plate (Greiner) at 30°C in assay buffer (see above). The respective ligand was diluted in Master Mix (see above) and binding kinetics were initiated by the addition of 10 nmol/L KDAC8. The release of fluorogenic AMC substrate was observed in a microplate reader (PHERAstar Optima, BMG Labtech) at 450 nm (Ex: 350 nm). All solutions were pre-incubated at 30°C. Slopes were calculated for the early-stage (1,000 s to 2,000 s) and the late-stage (5,000 s to 6,000 s) using linear regression. The relative KDAC8 activity in the presence of different inhibitor concentrations was calculated as the ratio of the late-stage and early-stage slopes and normalized to free KDAC8 enzyme.

### Determination of thermal stability

By utilizing a QuantStudio™ 5 real-time quantitative polymerase chain reaction (qPCR) system (Thermo Fisher Scientific), protein melting curves with SYPRO^®^ orange as fluorescence dye were generated. The samples contained 12 µmol/L KDAC8, a 10-fold concentration of SYPRO^®^ orange fluorescence dye (Sigma-Aldrich^®^, stock solution: 5,000-fold), and 100 µmol/L of the indicated compound in assay buffer (see above). A DMSO control without ligand was utilized as a positive control. After mixing the components, the samples were incubated at 30°C for 1 h in the real-time qPCR system to assure binding equilibrium. The heat gradient for the melting curves was 0.015°C/s, the fluorescence was measured at 623 nm (Ex: 580 nm). The fluorescence signal was plotted against the temperature. The melting point was calculated as the point of inflection of the first derivative using protein thermal shift software (Thermo Fisher Scientific).

## Results

### Development and validation of the continuous KDAC8 activity assay

**Figure 2:**
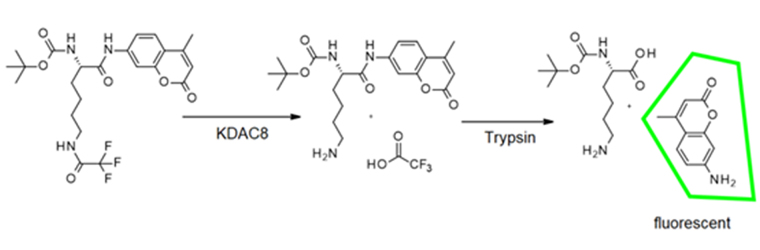
KDAC8 assay principle. The assay can be performed as a one-step or two-step assay

Usually, KDAC8 activity assays are performed in two subsequent steps, to avoid interference between the trypsin used in the second step and the enzyme activity of KDAC8 (Figure 2). However, if inhibitors shall be classified according to their binding kinetics, a continuous enzyme activity assay is required, which generates linear progress curves for the enzyme reaction over a long period time, and in the presence of preferably low enzyme concentration [17].

As a starting point for the development of a KDAC8 continuous assay, a two-step endpoint assay was used, which was established in our group [15]. During the first step of this assay the substrate Boc-Lys(TFA)-AMC is deacetylated by the KDAC enzyme. With the addition of a developer solution (a potent KDAC inhibitor and trypsin), KDAC activity is terminated and the deacetylated substrate is cleaved by trypsin (Figure 2).

This releases the AMC fluorophore, the signal intensity of which correlates to KDAC activity up to the time of addition of the developer solution. In the continuous KDAC8 assay, all components of the two-step endpoint assay are mixed at the beginning and the KDAC activity can be measured in a plate reader as the release of AMC over time. Range-finding experiments were carried out with varying trypsin concentrations and fixed KDAC8 and substrate concentration of 10 nmol/L and 20 µmol/L, respectively (Figure S1A). As expected, higher trypsin concentrations resulted in higher product release. However, trypsin is a serine protease and may also attack KDAC8 and even itself, which can result in lower product formation. This effect is noticeable at high trypsin concentrations (5 mg/mL). Nevertheless, trypsin concentrations between 0.078 mg/mL and 0.16 mg/mL exhibit little to none of these undesirable effects resulting in an essentially linear behavior over the measured time frame of 125 min. We chose a trypsin concentration of 0.1 mg/mL for the next experiment with varying KDAC8 concentrations. As expected, higher KDAC8 concentrations result in higher product formation (Figure S1B). The 5-fold higher product release rate of 50 nmol/L KDAC8 compared to 10 nmol/L KDAC8 also demonstrates that the product release rate is not limited by trypsin activity. Therefore, 10 nmol/L KDAC8 were used in the continuous assay.

### Michaelis-Menten kinetics

**Figure 3:**
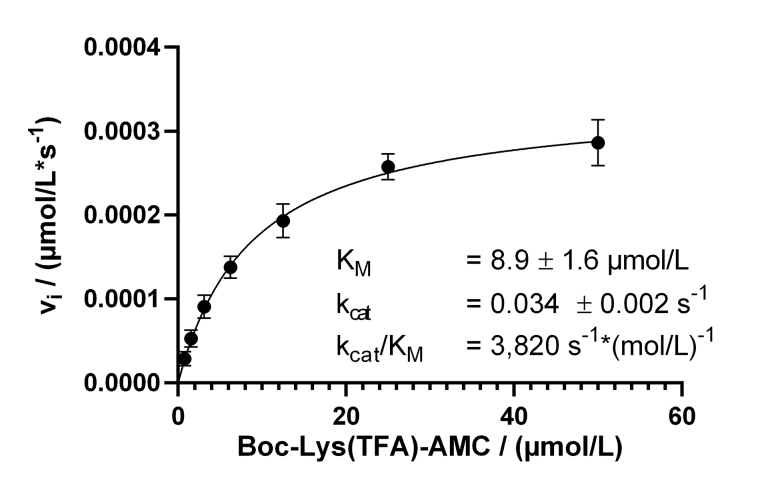
Michaelis-Menten kinetics of KDAC8 using the continuous enzyme activity assay. Parameters are provided as means ± standard deviations, *n* = 3. K_M_: Michaelis constant

With our now established continuous KDAC assay, enzyme kinetic experiments were performed to determine Michaelis-Menten parameters with substrate concentrations ranging from 780 nmol/L to 50 µmol/L. Over a while of 125 min, the substrate conversion ranged from 3% for 50 µmol/L substrate to 20% for 780 nmol/L substrate. The substrate conversion was quantified using a calibration curve of free AMC (Figure S2A). The resulting Michaelis-Menten plot and the obtained parameters are shown in Figure 3. Progress curves are also provided (Figure S2B).

The calculated K_M_ value of 8.9 µmol/L was lower as previously determined in a MAL buffer system [137 mmol/L NaCl, 50 mmol/L Tris-HCl, 2.7 mmol/L KCl, 1 mmol/L MgCl_2_, 1 mg/mL bovine serum albumin (BSA), pH 8.0]. To clarify this discrepancy, the Michaelis-Menten parameters were determined in MAL buffer using the continuous KDAC activity assay. A K_M_ value of 31 µmol/L was obtained, which was 3.5 times higher compared to the same assay carried out in assay buffer. Interestingly, the k_cat_ with a value of 0.18/s was higher in the MAL buffer system (Figure S3). The main difference between MAL buffer and assay buffer is the lower ionic strength of the assay buffer, and the addition of BSA to reduce surface adsorption. This observation indicates a major role of ionic interactions during substrate recognition by KDAC8, which is disturbed by the high salt concentration of MAL buffer resulting in significantly lower substrate affinity in the MAL buffer system. However, high ionic strength seems to be beneficial for substrate turnover.

### Dose-response curves of KDAC8 inhibitors with different binding kinetics and mode of action

**Figure 4:**
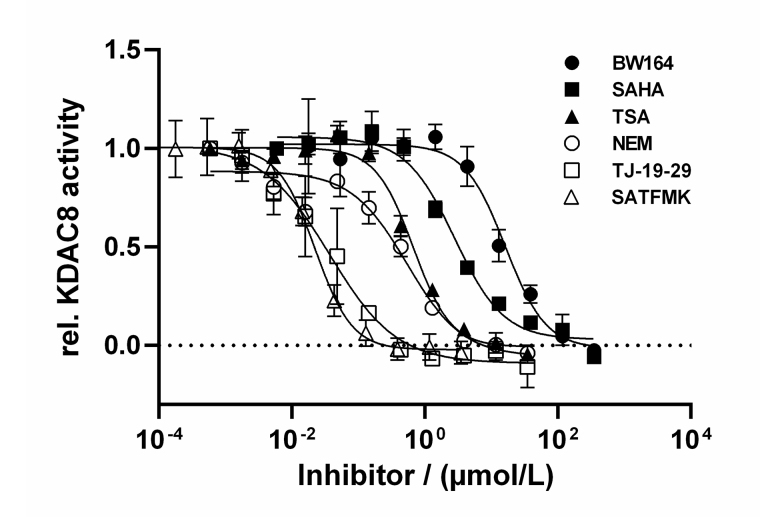
Dose-response curves of indicated inhibitors against KDAC8. Data represent means and standard deviations, *n* = 3

By using the newly established continuous assay, the affinity of six distinct KDAC8 inhibitors were determined, which were representative for fast (SAHA, TSA, BW164) or slow reversible binding (SATFMK) [18] or covalent inactivating (NEM, TJ-19-29) [19]. Obtained IC_50_-values were compared with those from our two-step endpoint assay (Figure 4, Table 1). Since two enzymes are utilized in the continuous KDAC8 assay (KDAC8 and trypsin), it is paramount to validate that the inhibitors only inhibit KDAC8 activity and not trypsin activity. Trypsin activity was verified not to be influenced by tested inhibitors at any applied concentration (Figure S4). Even NEM and TJ-19-29, both known cysteine modifiers, showed no effect against trypsin indicating that targeted cysteines are not essential for trypsin activity. With the continuous KDAC8 assay, IC_50_ values could be determined in a range between 17 µmol/L ± 4 µmol/L and 21 nmol/L ± 5 nmol/L for BW164 and SATFMK, respectively (Figure 4, Table 1). The fitted IC_50_ values of the continuous assay exhibit acceptable experimental errors and are in overall agreement with IC_50_ data of the two-step endpoint assay (Table 1, Figure S5) validating the suitability of the continuous KDAC8 assay to determine correct IC_50_-values from initial rates of progress curves for enzyme reaction after 60 min pre-incubation time.

**Table 1:** Summary of affinity data for tested KDAC8 inhibitors

**Inhibitor**	**IC_50_ (µmol/L)** **Continuous assay**	**IC_50_ (µmol/L)** **Two-step assay**	**SC_50_ (µmol/L)** **Stabilization curves**	**ΔT_m_ (°C)**	**Inhibition mode**
TSA	0.70 ± 0.16	0.30 ± 0.07	0.50 ± 0.11	11.96 ± 0.06	Fast reversible binding
SAHA	3.6 ± 0.9	1.9 ± 0.3	3.2 ± 0.9	8.50 ± 0.06	Fast reversible binding
BW164	17 ± 4	4.2 ± 0.6	31 ± 15	7.17 ± 0.06	Fast reversible binding
SATFMK	0.021 ± 0.005	0.021 ± 0.004	-	16.85 ± 0.18	Slow reversible binding (induced fit)
TJ-19-29	0.030 ± 0.009	0.12 ± 0.04	-	4.4 ± 0.3	Covalent inactivation
NEM	0.32 ± 0.09	0.78 ± 0.18	-	4.58 ± 0.26	Covalent inactivation

SC_50_ is the concentration of half-maximal KDAC8 stabilization and ΔT_m_ indicates the thermal stabilization of KDAC8 in the presence of 100 µmol/L inhibitor. SC_50_: ligand concentration, with half-maximal stabilization of enzyme activity; -: none

### Time-dependent IC_50_ values

**Figure 5:**
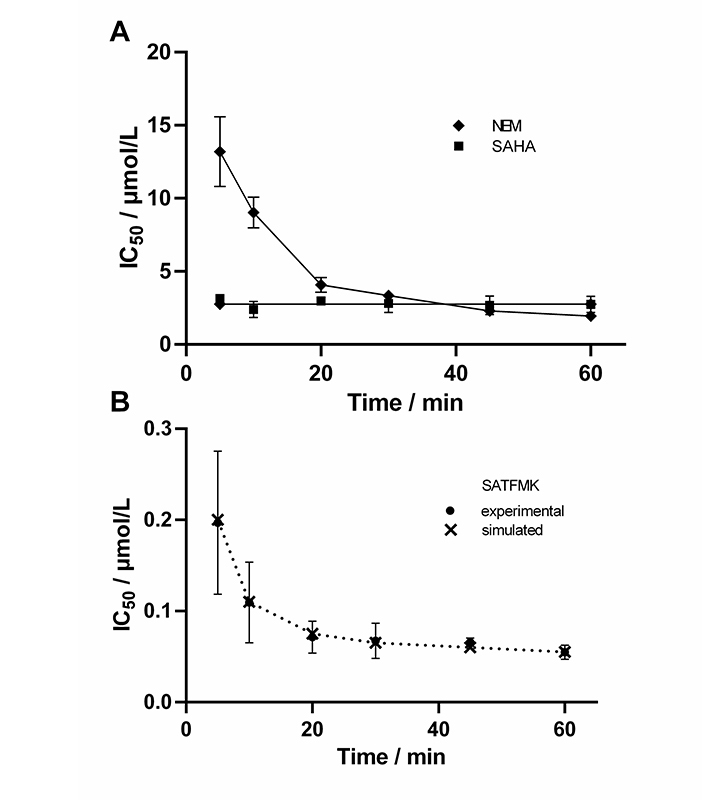
Time-dependent IC_50_-values of indicated inhibitors. For SATFMK (B) also simulated data are shown (×), which were calculated based on previously determined kinetic parameters [18]

In the next step, it was verified that the continuous KDAC8 activity assay is sensitive enough to determine time-dependent IC_50_ values. For this reason the well-known fast reversible inhibitor SAHA, the known covalent cysteine modifier NEM, and the slow binding inhibitor SATFMK (Figure 5) were tested. As expected, SAHA shows no time-dependent change in IC_50_-values confirming its fast-binding behavior. Furthermore, NEM exhibits significant changes in IC_50_-values over time indicating slow inactivation of KDAC8 due to covalent modification of functional cysteine residues. SATFMK was characterized as a slow-binding reversible inhibitor, which binds according to a two-step induced-fit binding mechanism [18]. The IC_50_-values for SATFMK against KDAC8 show a similar time-dependent behavior comparable to the covalent inactivator NEM. These observations are in excellent agreement with data simulated based on previously reported kinetic parameters (Figure 5B and S6) [18]. Thus, applying different pre-incubation times between KDAC8 and inhibitors, the continuous KDAC8 activity assay is capable to distinguish between fast reversible and slow binding or inactivating inhibitors via the determination of time-dependent IC_50_-values.

### Fast reversible KDAC8 inhibitors stabilize KDAC8 activity

**Figure 6:**
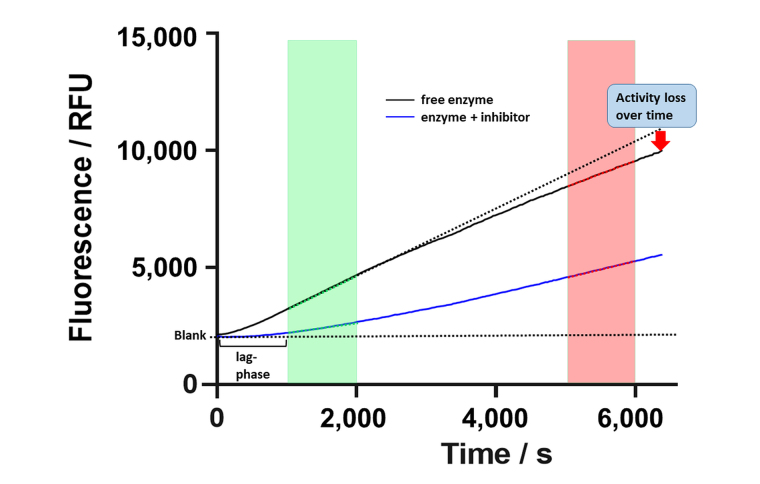
Schematic progress curve of continuous KDAC8 assay using Boc-Lys(TFA)-AMC substrate. Progress curves for free enzyme and enzyme in the presence of inhibitor are shown as black and blue solid lines. Dotted lines indicate ideal behavior for a fully inhibited or free enzymes. The green and red areas denote early and late-stage reaction times, respectively. The activity of free enzyme decreases slightly over time indicated by gradual deviation from linearity. RFU: relative fluorescence unit

Aiming at a more quantitative classification of KDAC8 inhibitors, the continuous KDAC8 assay was performed in the presence of different inhibitor concentrations. It is important to note that the inhibitor and the substrate plus trypsin were pre-mixed and the reaction was initiated by the subsequent addition of KDAC8. Although all components were pre-tempered to the desired temperature, it was not possible to completely eliminate the rather long lag phase of roughly 10 min. Although the continuous KDAC8 assay was optimized for linear behavior for the longest possible period, KDAC8 still showed a minor loss of activity over longer reaction times (Figure 6).

**Figure 7:**
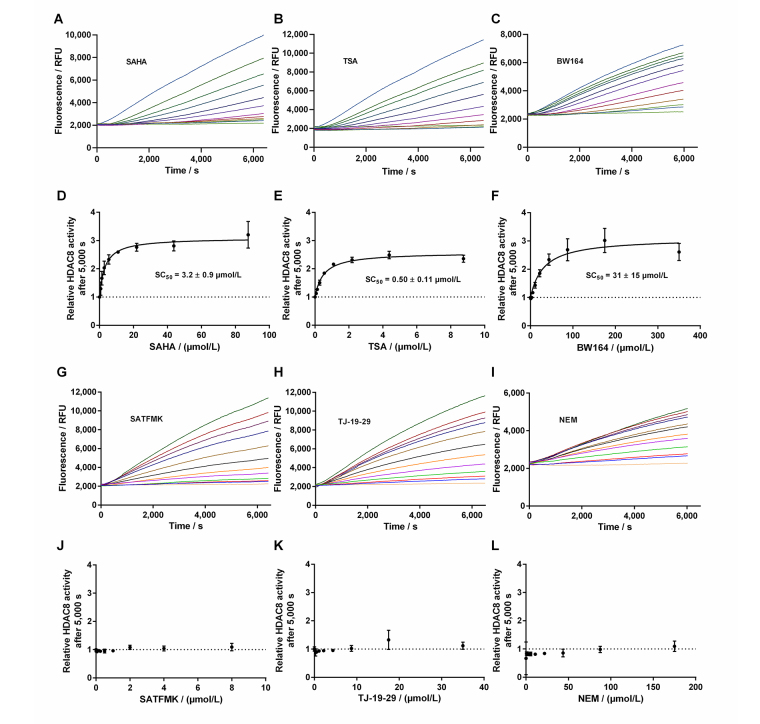
Progress curves of KDAC8 activity in the presence of different inhibitor concentrations. A. Progress curves in the presence of SAHA; B. progress curves in the presence of TSA; C. progress curves in the presence of BW164; D. relative KDAC8 activity after 5,000 s reaction time as a function of SAHA concentration; E. relative KDAC8 activity after 5,000 s reaction time as a function of TSA concentration; F. relative KDAC8 activity after 5,000 s reaction time as a function of BW164 concentration; G. progress curves in the presence of SATFMK; H. progress curves in the presence of TJ-19-29; I. progress curves in the presence of NEM; J. relative KDAC8 activity after 5,000 s reaction time as a function of SATFMK concentration; K. relative KDAC8 activity after 5,000 s reaction time as a function of TJ-19-29 concentration; L. relative KDAC8 activity after 5,000 s reaction time as a function of NEM concentration

This loss was attributed to the intrinsic stability of KDAC8 and/or slow gradual proteolysis by trypsin, which is required to generate the fluorescence signal. Unfortunately, the lag phase prevented the analysis of fast binding events and the gradual loss of enzyme activity over time complicated the straightforward analysis of binding kinetics of inhibitors according to Copeland [17]. Nevertheless, it was possible to obtain a robust and meaningful readout by calculating the ratio of slopes in the late stage (Figure 6, red area) and the early stage (Figure 6, green area) as a measure of relative KDAC8 activity after 5,000 s in the absence or in the presence of different inhibitor concentrations. Slopes within the early and late stages of the enzyme reaction were almost perfectly linear over 1,000 s with coefficients of determination, *r*^2^, equal to or greater than 0.999. The relative KDAC8 activity after 5,000 s was analyzed as a function of inhibitor concentration (Figure 7). Most interestingly, all known fast reversible inhibitors of the set of tested compounds, SAHA, TSA, and BW164, seem to stabilize KDAC8 because the relative KDAC8 activity after 5,000 s in the presence of these compounds is considerably higher than for free KDAC8 (Figure 7D–F). Although having different potencies ranging from IC_50_ = 0.7 µmol/L to 17 µmol/L, all fast reversible inhibitors increase the relative KDAC8 activity after 5,000 s reaction time by a factor of about 2.5 to 3. This indicates that the compounds not only prevent the loss of KDAC8 activity that is observed for the free enzyme but rather enhance the activity of KDAC8 regardless of affinity or chemical structure. Moreover, when fitting the stabilization curves in Figure 7D–F to a simple binding isotherm, the concentration of half-maximal stabilization, SC_50_, correlates nicely with independently determined IC_50_-values using the continuous KDAC8 assay after 60 min pre-incubation time (Pearson *r* = 0.9962, Table 1). This close relationship suggests that the stabilization of KDAC8 is intimately linked to fast reversible binding to the active site. In contrast, neither covalent cysteine modifiers NEM or TJ-19-29, nor the slowly reversible KDAC8 inhibitor SATFMK can stabilize KDAC8 activity with regard to free enzyme (Figure 7J–L). The structural stabilization of KDAC8 upon binding of tested inhibitors was also determined by thermal shift experiments (Figure S7). KDAC8 turns out to be a rather flexible protein with relatively low thermal stability (T_m_ = 42.67°C ± 0.06°C). Binding of reversible inhibitors increases the T_m_ tremendously by 7°C to 17°C (Table 1). In contrast, covalent modifiers NEM and TJ-19-29 stabilize native KDAC8 only by 4–5°C. Thermal stabilization should not be confused with functional stabilization as described before and assessed by using the continuous KDAC8 assay. Thermal stabilization of KDAC8’s structure upon binding of SATFMK or covalent cysteine modification does not correlate with the preservation of KDAC8 activity.

## Discussion

Usually, two-step enzyme activity assays are used to monitor the activity of zinc-containing KDACs. The first step consists of the deacetylation reaction itself, mostly applying an artificial substrate containing an acetylated or trifluoroacetylated lysine residue. In the second step, a mixture of a highly active inhibitor and trypsin is applied to stop the deacetylation reaction and develop the signal by releasing a fluorescent dye from the deacetylated substrate. The separation of the deacetylase and detection reaction was established because trypsin is in principle capable of degrading the target enzyme or even itself. This type of endpoint assay is useful to determine IC_50_-values of inhibitors and has also been used to screen compound libraries for new inhibitory chemotypes [20]. However, the two-step assay format has its limitations, when aiming at more detailed information about the mechanism of inhibition. In the pharmaceutical context, it is of great importance to classify inhibitory hit compounds according to their mode of action. Using continuous enzyme activity assays, it is possible to measure the binding kinetics of inhibitors, and also to distinguish between different inhibition mechanisms, be it simple one-step or two-step binding, reversible inhibition, or covalent inactivation [17, 21]. Several specialized substrates have been developed, which enable continuous KDAC assays, and avoid the above-mentioned developer enzyme trypsin [22, 23]. In this study, the established two-step assay for KDAC8 using the simple commercially available Boc-Lys(TFA)-AMC substrate was developed further towards a one-step assay in the presence of both, KDAC8 and trypsin. The one-step or continuous KDAC8 assay was validated by determining Michaelis-Menten kinetics and comparing IC_50_-values for diverse inhibitors with IC_50_-values of the established two-step assay. For the low-salt assay buffer, a K_M_ of (8.9 ± 1.6) µmol/L, and a k_cat_ = (0.034 ± 0.002)/s was determined. At higher ionic strength a K_M_ value of 31 µmol/L and an almost six-fold k_cat_ = 0.18/s, were observed, in accordance with previous results [24]. The K_M_ value as well as the k_cat_ greatly depends on ionic strength indicating ionic interactions between the substrate and KDAC8. The effect on the k_cat_ is harder to explain and might be an indirect effect on the conformational flexibility of KDAC8, which is required for catalytic activity [25]. The fast-reversible inhibitors TSA, SAHA, and BW164 were selected to cover an IC_50_ range from 0.7 µmol/L to 17 µmol/L. SATFMK was chosen because this inhibitor has a high binding affinity (IC_50_ = 0.021 µmol/L) and a slow two-step induced fit binding mechanism. In addition, two more covalent inactivators, NEM and TJ-19-29, were selected, which have a tenfold difference in IC_50_ value in the continuous assay. IC_50_-values obtained for the continuous and the established two-step KDAC8 activity assay are in acceptable agreement over a wide range of three orders of magnitude from (21 ± 5) nmol/L (SATFMK) to (17 ± 4) µmol/l (BW164) (Table 1). Therefore, the continuous KDAC8 assay is suitable for equilibrium affinity determinations. To demonstrate the capability of the assay to acquire a slow kinetic inhibitory effect of active compounds on KDAC8 activity, IC_50_-values were determined for different pre-incubation times (Figure 5). Due to technical limitations, the shortest pre-incubation time was 5 min, which does not allow to acquire faster binding kinetics. Nonetheless this setup enabled the detection of slow covalent inactivation of KDAC8 by NEM, and slow binding of reversible inhibitor SATFMK to KDAC8, since the corresponding IC_50_-values decreased with increasing pre-incubation time (Figure 5). The observed slow inhibition kinetics of SATFMK is in agreement with the previous determination of binding kinetics and mechanism of SATFMK to KDAC8 [18]. In contrast, fast binding inhibitor SAHA showed an unchanged IC_50_-value independent of pre-incubation time, because binding equilibrium was established in less than 5 min, also in accordance with previously reported binding kinetic data [18]. For the continuous KDAC8 assay, conditions were found, where the progress curve of the enzyme reaction is almost linear for more than 1 h, although a gradual loss of enzyme activity could not be completely prevented (Figure 7). The slight loss of enzyme activity is most likely caused by partial hydrolysis by trypsin or inherent instability of KDAC8, which was determined by thermal shift experiments (T_m_ = 42.67°C ± 0.06°C). It is noteworthy that the enzyme activity assays were performed at 30℃. The observed minor deviation from linearity over a long period time (5,000 s), as well as the rather long lag phase of about 10 min after the start of the reaction, complicated a rigorous analysis of the progress curve according to Copeland [17]. Despite the sobering results in terms of long-term linearity, the progress curves of KDAC8 activity were measured in the presence and absence of known KDAC8 inhibitors with different chemical structures and modes of action to assess the potential for categorizing agents according to their mechanism of action. All fast reversible inhibitors not only preserved but rather enhanced the relative KDAC8 activity after 5,000 s with respect to free enzyme by a factor of 2.5 to 3 (Figure 7D–F), although having different IC_50_-values ranging from 0.7 µmol/L (TSA) to 17 µmol/L (BW164) (Table 1). The data could be fitted to a simple binding isotherm providing the concentration of half-maximal stabilization, SC_50_. Strikingly, these SC_50_-values show excellent correlation with IC_50_-values of the continuous KDAC8 assay (Pearson *r* = 0.9962) (Table 1) suggesting that enzyme activity is intimately linked to KDAC8 interaction with fast reversible inhibitors. Earlier work already showed that some inhibitors could act as chaperones and rescue misfolded protein conformations [26]. Such an effect was previously described for SAHA, which is capable of stabilizing KDAC11 [27]. The authors suggest adding the IC_50_ concentration of SAHA to KDAC11 assays to achieve robust conditions for hit discovery in high-throughput screening campaigns. The pronounced 2.5-fold to 3-fold enhancement of relative KDAC8 activity lets us hypothesize that fast and reversible binding of inhibitors is a feasible mechanism by which misfolded conformations of KDAC8 can be repaired. In contrast, slowly reacting covalent inactivators irreversibly inactivate KDAC8, while the remaining free KDAC8 should slowly lose activity similar to KDAC8 in the absence of an inhibitor. This expectation is confirmed for TJ-19-29 and NEM, which do not preserve the relative KDAC8 activity over a long reaction time (Figure 7K and L). Interestingly, slow-binding reversible inhibitor SATFMK is also not capable of preserving or enhancing the enzyme activity of KDAC8 (Figure 7J). This might be explained by the very slow overall dissociation rate, which is caused by a two-step binding mechanism of SATFMK involving an induced fit [18]. The long residence time on the KDAC8 target prevents multiple binding and release of free KDAC8 molecules, which is supposed to enable effective repair of misfolded KDAC8 conformations. Since KDAC8 is thermally rather unstable, it is not unexpected that all reversible inhibitors showed drastic thermal shifts and stabilization of KDAC8 by 7°C to 17°C (Table 1). The example of SATFMK demonstrates that structural stabilization of KDAC8 upon equilibrium binding is not correlated with the ability to recover KDAC8 activity, because the repair of misfolded conformations is a dynamic process. Thermal stabilization is a consequence of shifting the binding equilibrium toward the KDAC8-inhibitor complex. Therefore, slow dissociating inhibitors may cause particularly high thermal stabilization, because the labile-free enzyme is only slowly released from the complex.

Compared with routinely applied two-step assays, the continuous KDAC8 assay provides not only comparable equilibrium affinity data but also yields additional information about the mode of action of inhibitory compounds. The continuous KDAC8 assay is easier to perform and has fewer pipetting steps. The assay is homogeneous and thus suitable for one-dose high-throughput screening of large compound libraries. By applying a long pre-incubation time of 60 min even slow binders will be identified by analyzing the slopes within the early stage of the progress curve. Analyzing the ratio of slopes of the late and early stages from the same data set provides additional information about stabilizing effects of hit compounds on KDAC8, which is a strong indication of a fast and reversible inhibitor. Hit compounds that do not stabilize could be slowly reversible binders, covalent inactivators, or have a different mode of action such as allosteric inhibition. Therefore, the continuous KDAC8 assay can be used for early high-throughput classification of active chemotypes in terms of mode of action. In a confirmatory assay, the IC_50_-values can be determined using the same assay format. Moreover, a modified continuous KDAC8 assay with different pre-incubation times can be used to detect slow inhibitory active compounds. If required, further differentiation between slowly dissociating reversible inhibitors and covalent inactivators can be carried out using rapid dilution or dialysis experiments.

## Abbreviations

AMC: 7-amino-4-methyl-coumarin
Boc-Lys(TFA)-AMC: tert-butyl *N*-[(1S)-1-[(4-methyl-2-oxo-chromen-7-yl)carbamoyl]-5-[(2,2,2-trifluoroacetyl)amino]pentyl]carbamate
BW164: 2,2-diphenylethanehydroxamic acid
IC_50_: inhibitor concentration that inhibits 50% of enzyme activity
k_cat_: turnover number
KDAC8: histone deacetylase 8
KDACs: lysine deacetylases/deacylases
K_M_: Michaelis constant
NEM: *N*-ethylmaleimide
SAHA: suberoylanilide hydroxamic acid
SATFMK: 9,9-trifluoro-8-oxo-*N*-phenyl-nonanamide
SC_50_: ligand concentration, with half-maximal stabilization of enzyme activity
SUMO: small ubiquitin-like modifier
TJ-19-29: 3-chloro-5-[2-(4-nitrophenyl)ethanesulfonyl]-1,2,4-thiadiazole
TSA: trichostatin A

## Supplementary materials

The supplementary materials for this article is available at: https://www.explorationpub.com/uploads/Article/file/1002144_sup_1.pdf.

## Declarations

### Availability of data and materials

All experimental information is contained in the manuscript and supporting information.

### Copyright

© The Author(s) 2023.
