# Supplementary material for: Continuous enzyme activity assay for high-throughput classification of histone deacetylase 8 inhibitors
Source: Explor Target Antitumor Ther. 2023 Jun 30;4(3):447–59. doi: 10.37349/etat.2023.00144 (PMC10344891; doi:10.37349/etat.2023.00144)
Supplement: Supplementary file 1 [file etat-04-1002144-s001.pdf]

Supplementary materials

Continuous enzyme activity assay for high-throughput classification of histone deacetylase 8 inhibitors

Markus Schweipert, Anuja Amurthavasan, Franz-Josef Meyer-Almes*

Chemical Engineering and Biotechnology, University of Applied Sciences Darmstadt, 64295 Darmstadt, Germany

***Correspondence:** Franz-Josef Meyer-Almes, Chemical Engineering and Biotechnology, University of Applied Sciences Darmstadt, Haardring 100, 64295 Darmstadt, Germany. franz-josef.meyer-almes@h-da.de

Content:

Figure S1: Range Finding for One Step KDAC8 enzyme activity assay

Figure S2: Michaelis Menten Progress Curves in Assay Buffer

Figure S3: Michaelis Menten plot of KDAC8 in MAL buffer

Figure S4: Dose response curves of inhibitors against trypsin

Figure S5: Dose response curves of inhibitors in two-step assay with KDAC8

Figure S6: Thermal Shift Data of KDAC8 inhibitors

Figure S7: Simulated binding kinetics of SATFMK to KDAC8 using COPASI

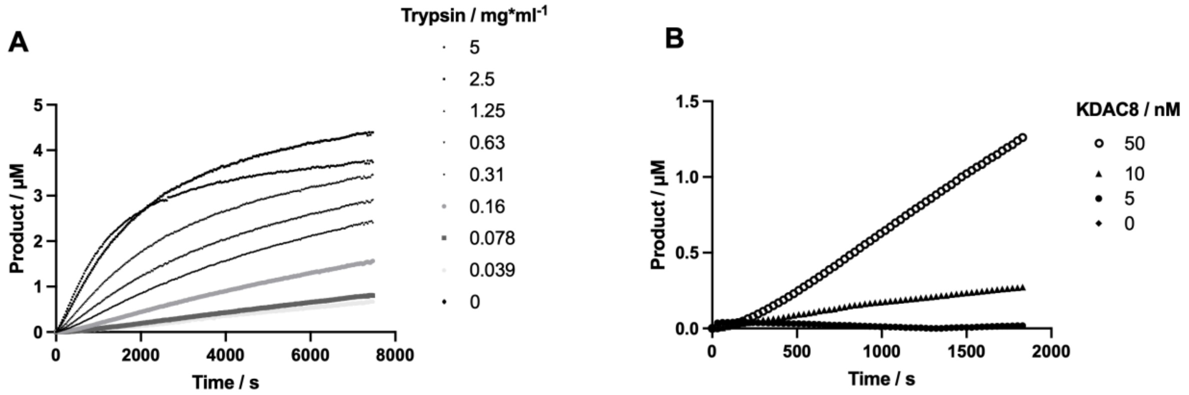

Figure S1. Range Finding for One Step KDAC8 enzyme activity assay. (A) The one step KDAC8 enzyme activity assay was performed using 10 nM KDAC8, 20 µmol/L Boc-Lys(TFA)-AMC and the indicated trypsin concentrations. (B) The one step KDAC8 enzyme activity assay was performed with 0.1 mg/mL trypsin, 20 µmol/L substrate and the indicated KDAC8 concentrations

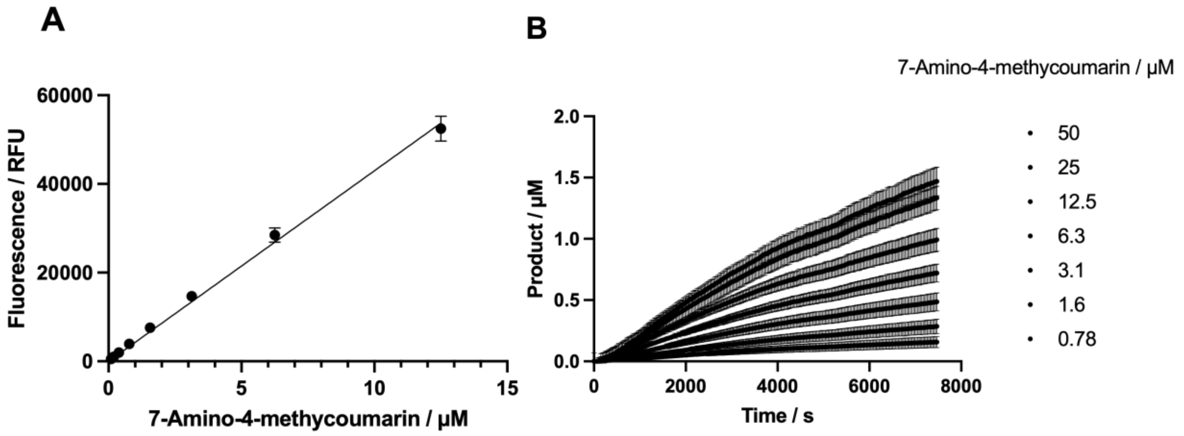

Figure S2. Michaelis Menten Progress Curves in Assay Buffer. (A) Calibration curve to transform the fluorescence signal into product concentration. (B) Progress curves using indicated substrate concentrations in order to determine Michaelis-Menten parameters. N=4.

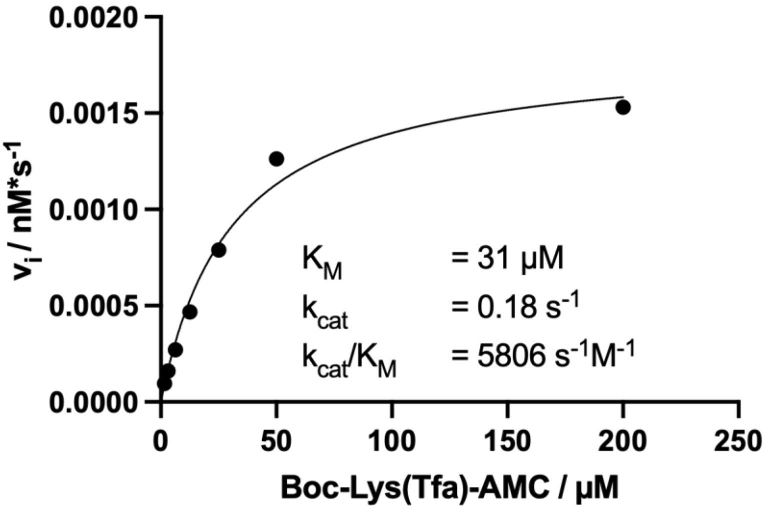

Figure S3. Michaelis Menten plot of KDAC8 in MAL buffer

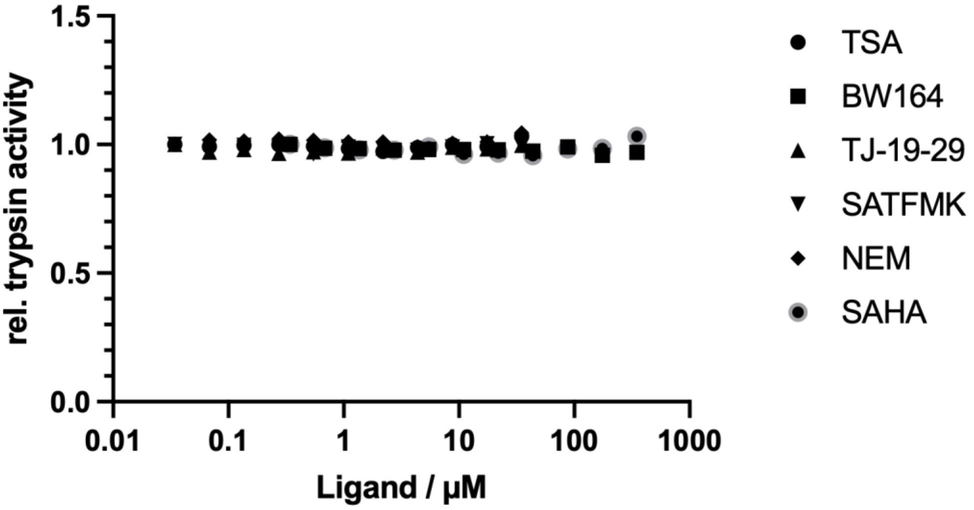

Figure S4. Dose response curves of inhibitors against trypsin

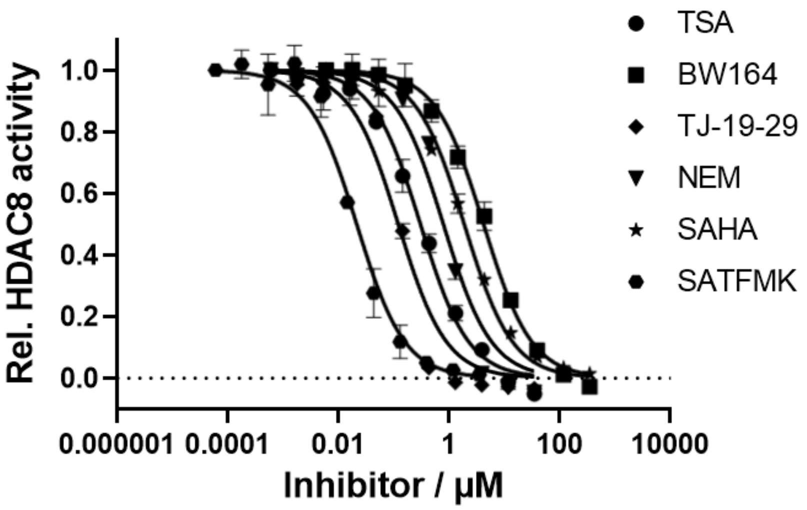

Figure S5. Dose response curves of inhibitors in two-step assay with KDAC8. N=3.

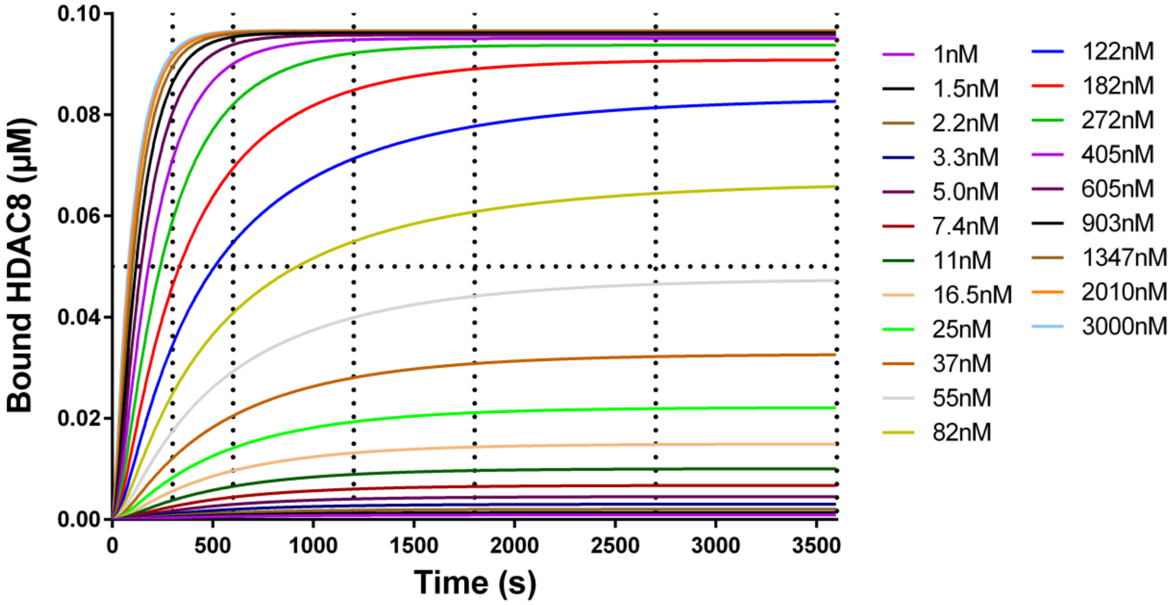

Figure S6. Simulated binding kinetics of SATFMK to KDAC8 using COPASI

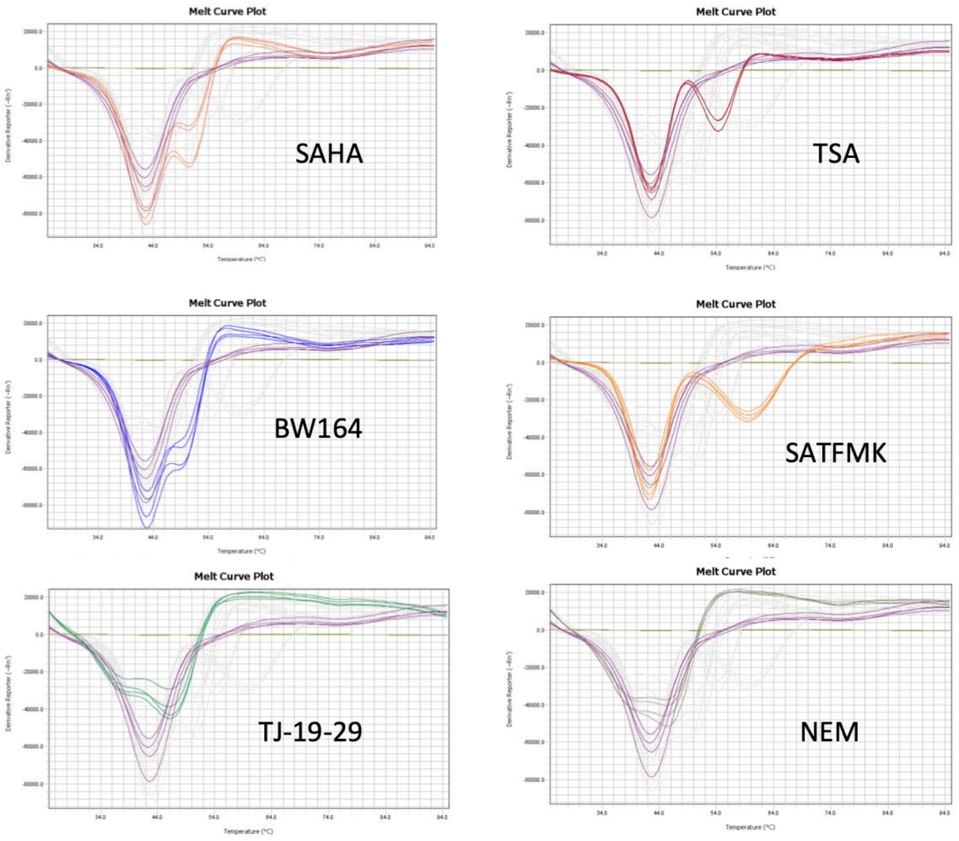

Figure S7. Thermal Shift Data of KDAC8 inhibitors. First derivative of fluorescence signal is plotted against temperature. Purple is free KDAC8. Straight olive line is SYPRO Orange fluorescence dye without KDAC8 (negative control). Each replicate is plotted, n=4
